# *N*,*N*-Dimethylpyridin-4-aminium 1-phenyl­cyclo­pentane-1-carboxyl­ate monohydrate

**DOI:** 10.1107/S1600536811015200

**Published:** 2011-04-29

**Authors:** Guangwen He, Srinivasulu Aitipamula, Pui Shan Chow, Reginald B. H. Tan

**Affiliations:** a Institute of Chemical and Engineering Sciences, A*STAR (Agency for Science, Technology and Research), 1 Pesek Road, Jurong Island, Singapore 627833; b Department of Chemical & Biomolecular Engineering, National University of Singapore, 4 Engineering Drive 4, Singapore 117576

## Abstract

The cation of the title salt, C_7_H_11_N_2_^+^·C_12_H_13_O_2_^−^·H_2_O, is planar (r.m.s. deviation = 0.0184 Å). In the crystal, the cation, anion and water mol­ecule are linked by O—H⋯O and N—H⋯O hydrogen bonds, forming a chain running along the *a* axis.

## Related literature

For the structure of 4-dimethyl­amino­pyridine, see: Ohms & Guth (1984 ▶). For the structure of 1-phenyl­cyclo­pentane-1-carb­oxy­lic acid, see: Margulis (1975 ▶). For recent mol­ecular co-crystals and salts of 4-dimethyl­amino­pyridine, see: Dastidar *et al.* (1993 ▶). For recent mol­ecular co-crystals of 1-phenyl­cyclo­pentane-1-carb­oxy­lic acid, see: He *et al.* (2010 ▶, 2011 ▶). For comparative bond dimensions in pyridinium carboxyl­ates, see: Kumar *et al.* (2009 ▶).
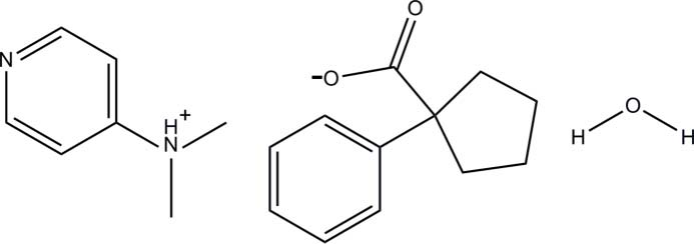


## Experimental

### 

#### Crystal data

C_7_H_11_N_2_^+^·C_12_H_13_O_2_^−^·H_2_O
*M* *_r_* = 330.42
Monoclinic, 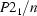

*a* = 6.1666 (12) Å
*b* = 18.206 (4) Å
*c* = 15.702 (3) Å
β = 97.33 (3)°
*V* = 1748.4 (6) Å^3^
*Z* = 4
Mo *K*α radiation
μ = 0.09 mm^−1^
*T* = 110 K
0.44 × 0.33 × 0.22 mm

#### Data collection

Bruker APEXII diffractometer
Absorption correction: multi-scan (Blessing, 1995 ▶) *T*_min_ = 0.964, *T*_max_ = 0.982
12519 measured reflections
4233 independent reflections
3945 reflections with *I* > 2σ(*I*)
*R*_int_ = 0.017

#### Refinement

*R*[*F* ^2^ > 2σ(*F* ^2^)] = 0.051
*wR*(*F* ^2^) = 0.139
*S* = 1.13
4233 reflections
231 parameters
3 restraints
H atoms treated by a mixture of independent and constrained refinement
Δρ_max_ = 0.23 e Å^−3^
Δρ_min_ = −0.21 e Å^−3^

### 

Data collection: *APEX2* (Bruker, 2007 ▶); cell refinement: *SAINT* (Bruker, 2007 ▶); data reduction: *SAINT*; program(s) used to solve structure: *SHELXS97* (Sheldrick, 2008 ▶); program(s) used to refine structure: *SHELXL97* (Sheldrick, 2008 ▶); molecular graphics: *X-SEED* (Barbour, 2001 ▶); software used to prepare material for publication: *SHELXTL* (Sheldrick, 2008 ▶) and *PLATON* (Spek, 2009 ▶).

## Supplementary Material

Crystal structure: contains datablocks I, global. DOI: 10.1107/S1600536811015200/ng5153sup1.cif

Structure factors: contains datablocks I. DOI: 10.1107/S1600536811015200/ng5153Isup2.hkl

Supplementary material file. DOI: 10.1107/S1600536811015200/ng5153Isup3.cml

Additional supplementary materials:  crystallographic information; 3D view; checkCIF report

## Figures and Tables

**Table 1:** Hydrogen-bond geometry (Å, °)

*D*—H⋯*A*	*D*—H	H⋯*A*	*D*⋯*A*	*D*—H⋯*A*
N1—H7⋯O2	0.94 (2)	1.72 (2)	2.6458 (15)	168 (2)
O3—H3⋯O2	0.87 (2)	1.93 (2)	2.7935 (14)	167 (2)
O3—H6⋯O1^i^	0.87 (2)	1.90 (2)	2.7634 (15)	169 (2)

Symmetry code: (i) 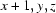.

**Fig. 1.**
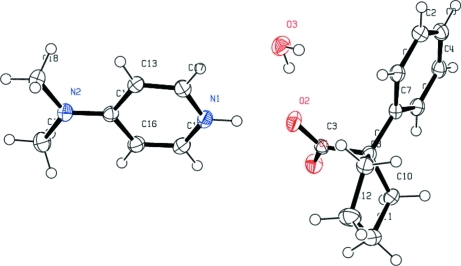
The molecular structures of 4-dimethylaminopyridine, 1-phenylcyclopentane-1-carboxylic acid and water, with atom labels and 50% probability displacement ellipsoids for non-H atoms.

**Fig. 2.**
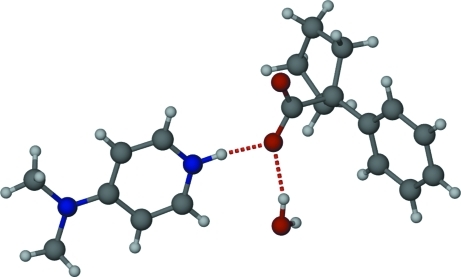
A supramolecular unit in the crystal structure of the title salt hydrate, featuring the O—H···O interaction between the carboxylate ion and the water molecule and the N—H···O interaction between the carboxylate ion and the pyridinium ions.

**Fig. 3.**
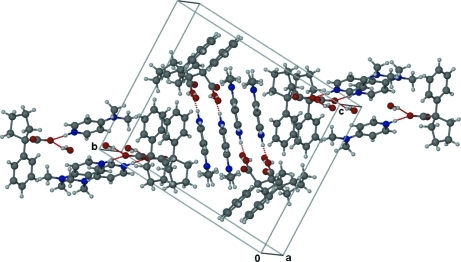
Part of the crystal structure of the title salt hydrate, showing the close packing of hydrogen bonded chains.

**Fig. 4.**
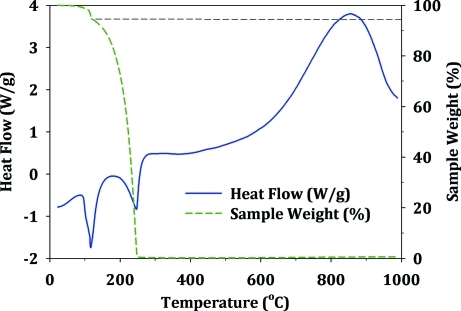
Profiles of heat flow and weight loss of the title salt hydrate determined by DSC and TGA, respectively.

## supplementary crystallographic information

### Comment

Substituted pyridines such as 4-dimethylaminopyridine was found to form binary salt hydrate with l-tartaric acid (Dastidar *et al.*, 1993). The authors have shown that this molecular complex possesses high nonlinear optical (NLO) effects, *viz.* second harmonic generation (SHG) in the crystalline state. In our previous work, we have demonstrated the formation of a salt and a cocrystal of a substituted pyridine, 2-aminopyridine, with 1-phenylcyclopropane-1-carboxylic acid and 1-phenylcyclopentane-1-carboxylic acid, respectively (He *et al.*, 2010; He *et al.*, 2011). Here we have selected 4-dimethylaminopyridine and 1-phenylcyclopentane-1-carboxylic acid as a model molecular pair.

The crystal structure of the title salt hydrate contains each one molecule of 4-dimethylaminopyridinium ion, 1-phenylcyclopentane-1-carboxylate ion, and water (Fig. 1). The title molecular complex is a salt rather than a cocrystal is evident by the proton transfer from the carboxylic acid to the pyridine nitrogen of the 4-dimethylaminopyridine, which was located in the difference Fourier map during the refinement cycles. Furthermore, the C—O/C=O bond distances (1.2412 (16) Å and 1.2700 (16) Å) and C—N—C angle of pyridine group (119.93 (12)°) are in well agreement with the corresponding distances/angles that are generally observed for a carboxylic acid-pyridine salts (Kumar *et al.*, 2009). In the crystal structure, the translation related 1-phenylcyclopentane-1-carboxylate ions are connected *via* water molecules involving O—H···O hydrogen bonds (Table 1) and generate infinite hydrogen bonded chains along the crystallographic *a*-axis (Fig. 2). The 4-dimethylaminopyridinium ion hydrogen bonded to one of the O atoms of the carboxylate ion *via* N—H···O hydrogen bond (Fig. 2). The hydrogen bonded chians close pack to build up the overall crystal structure (Fig. 3). A TGA experiment indicates an initial weight loss (*ca* 6%) upon heating (Fig. 4). This number matches with the water content in the title salt hydrate, implying that the resulting molecular complex is indeed a hydrate.

### Experimental

0.1224 g (1 mmol) of 4-dimethylaminopyridine (Alfa Aesar, 99%) and 0.1902 g (1 mmol) of 1-phenylcyclopentane-1-carboxylic acid (Alfa Aesar, 98%) and were dissolved into 7 ml of acetonitrile/water (90/10 *v*/*v*%) (acetonitrile, Fisher Scientific, HPLC; deionized water). Solution was then filtered through a 0.22 µm PTFE filter. Filtered solution was finally sealed with Parafilm and small holes were made to allow solvent to slowly evaporate. The colorless block-shaped crystal (0.44 × 0.33 × 0.22 mm) suitable for single-crystal X-ray diffraction (Rigaku Saturn 70 CCD area detector with Mo *K*_α_ radiation = 0.71073 Å at 50 kV and 40 mA) was collected after three day. TGA-DSC experiment of the resulting crystals was run using a TA Instrument SDT-TGA (SDT2960) at a ramping rate of 10 °C/min to 1000 °C.

### Refinement

A low-angle reflection, (011), whose intensity was strongly affected by the beam-stop, was omitted in the refinement cycles. H atoms bonded to N and O atoms were located in a difference map and allowed to ride on their parent atoms in the refinement cycles.The O—H bond distances and H—O—H angle of the water molecule were found to be deviating from the normal values. These were restrained using *DFIX* and DANG commands in the *SHELX*, and the deviations from the normal values are 0.04 (2), 0.02 (2) and 4 (2). The H atoms connected to C atoms were positioned geometrically and refined using a riding model.

### Crystal data

**Table:**

C_7_H_11_N_2_^+^·C_12_H_13_O_2_^−^·H_2_O	*F*(000) = 712
*M**_r_* = 330.42	*D*_x_ = 1.255 Mg m^−^^3^
Monoclinic, *P*2_1_/*n*	Mo *K*α radiation, λ = 0.71073 Å
Hall symbol: -P 2yn	Cell parameters from 5283 reflections
*a* = 6.1666 (12) Å	θ = 2.2–31.0°
*b* = 18.206 (4) Å	µ = 0.09 mm^−^^1^
*c* = 15.702 (3) Å	*T* = 110 K
β = 97.33 (3)°	Block, colorless
*V* = 1748.4 (6) Å^3^	0.44 × 0.33 × 0.22 mm
*Z* = 4	

### Data collection

**Table:**

Bruker APEXII diffractometer	4233 independent reflections
Radiation source: fine-focus sealed tube	3945 reflections with *I* > 2σ(*I*)
graphite	*R*_int_ = 0.017
ω scans	θ_max_ = 28.3°, θ_min_ = 2.9°
Absorption correction: multi-scan (Blessing, 1995)	*h* = −8→8
*T*_min_ = 0.964, *T*_max_ = 0.982	*k* = −21→23
12519 measured reflections	*l* = −20→20

### Refinement

**Table:**

Refinement on *F*^2^	Primary atom site location: structure-invariant direct methods
Least-squares matrix: full	Secondary atom site location: difference Fourier map
*R*[*F*^2^ > 2σ(*F*^2^)] = 0.051	Hydrogen site location: inferred from neighbouring sites
*wR*(*F*^2^) = 0.139	H atoms treated by a mixture of independent and constrained refinement
*S* = 1.13	*w* = 1/[σ^2^(*F*_o_^2^) + (0.0729*P*)^2^ + 0.4696*P*] where *P* = (*F*_o_^2^ + 2*F*_c_^2^)/3
4233 reflections	(Δ/σ)_max_ < 0.001
231 parameters	Δρ_max_ = 0.23 e Å^−^^3^
3 restraints	Δρ_min_ = −0.21 e Å^−^^3^

### Special details

**Table:**

Geometry. All e.s.d.'s (except the e.s.d. in the dihedral angle between two l.s. planes) are estimated using the full covariance matrix. The cell e.s.d.'s are taken into account individually in the estimation of e.s.d.'s in distances, angles and torsion angles; correlations between e.s.d.'s in cell parameters are only used when they are defined by crystal symmetry. An approximate (isotropic) treatment of cell e.s.d.'s is used for estimating e.s.d.'s involving l.s. planes.
Refinement. Refinement of *F*^2^ against ALL reflections. The weighted *R*-factor *wR* and goodness of fit *S* are based on *F*^2^, conventional *R*-factors *R* are based on *F*, with *F* set to zero for negative *F*^2^. The threshold expression of *F*^2^ > σ(*F*^2^) is used only for calculating *R*-factors(gt) *etc*. and is not relevant to the choice of reflections for refinement. *R*-factors based on *F*^2^ are statistically about twice as large as those based on *F*, and *R*- factors based on ALL data will be even larger.

### Fractional atomic coordinates and isotropic or equivalent isotropic displacement parameters (Å^2^)

**Table:**

	*x*	*y*	*z*	*U*_iso_*/*U*_eq_	
O3	0.46197 (17)	0.37281 (6)	0.40367 (7)	0.0322 (2)	
H3	0.337 (3)	0.3496 (12)	0.3979 (14)	0.062 (6)*	
H6	0.561 (3)	0.3390 (10)	0.4004 (13)	0.053 (6)*	
C1	−0.0970 (2)	0.21395 (7)	0.17867 (8)	0.0237 (3)	
H1	−0.2349	0.1936	0.1864	0.028*	
C2	0.3042 (2)	0.27379 (7)	0.15451 (8)	0.0270 (3)	
H2	0.4419	0.2941	0.1463	0.032*	
C3	−0.06856 (19)	0.25206 (7)	0.38099 (7)	0.0206 (2)	
C4	−0.0648 (2)	0.24247 (7)	0.09887 (8)	0.0261 (3)	
H4	−0.1802	0.2410	0.0526	0.031*	
C5	0.2731 (2)	0.24498 (7)	0.23453 (8)	0.0232 (3)	
H5	0.3897	0.2458	0.2803	0.028*	
C6	0.03032 (19)	0.18630 (7)	0.33520 (7)	0.0206 (2)	
C7	0.07135 (19)	0.21492 (7)	0.24761 (7)	0.0203 (2)	
C8	0.2357 (2)	0.15696 (7)	0.39066 (8)	0.0256 (3)	
H8A	0.3231	0.1256	0.3564	0.031*	
H8B	0.3283	0.1978	0.4160	0.031*	
C9	0.1353 (2)	0.27300 (7)	0.08674 (8)	0.0277 (3)	
H9	0.1567	0.2932	0.0326	0.033*	
C10	−0.1238 (2)	0.11900 (7)	0.33039 (8)	0.0269 (3)	
H10A	−0.2786	0.1346	0.3193	0.032*	
H10B	−0.0928	0.0852	0.2841	0.032*	
C11	−0.0764 (3)	0.08142 (8)	0.41878 (9)	0.0361 (3)	
H11A	−0.1933	0.0928	0.4545	0.043*	
H11B	−0.0680	0.0275	0.4120	0.043*	
C12	0.1442 (3)	0.11201 (9)	0.46066 (9)	0.0367 (3)	
H12A	0.1235	0.1436	0.5104	0.044*	
H12B	0.2450	0.0715	0.4807	0.044*	
O1	−0.26922 (15)	0.25454 (6)	0.38244 (6)	0.0300 (2)	
O2	0.06558 (15)	0.30076 (5)	0.41263 (6)	0.0302 (2)	
C13	0.1993 (2)	0.49016 (7)	0.62159 (8)	0.0232 (3)	
H13	0.3239	0.5208	0.6343	0.028*	
C14	0.0210 (2)	0.49587 (7)	0.67079 (8)	0.0227 (3)	
C15	−0.1584 (2)	0.40174 (7)	0.57900 (9)	0.0275 (3)	
H15	−0.2822	0.3714	0.5629	0.033*	
C16	−0.1610 (2)	0.44957 (7)	0.64561 (9)	0.0270 (3)	
H16	−0.2856	0.4519	0.6754	0.032*	
C17	0.1905 (2)	0.44045 (7)	0.55616 (8)	0.0247 (3)	
H17	0.3111	0.4367	0.5243	0.030*	
C18	0.2073 (2)	0.59162 (8)	0.76179 (9)	0.0325 (3)	
H18A	0.2461	0.6166	0.7105	0.049*	
H18B	0.3331	0.5634	0.7886	0.049*	
H18C	0.1658	0.6282	0.8026	0.049*	
C19	−0.1612 (3)	0.54665 (9)	0.78639 (10)	0.0383 (3)	
H19A	−0.1988	0.4973	0.8047	0.057*	
H19B	−0.2870	0.5680	0.7503	0.057*	
H19C	−0.1224	0.5777	0.8370	0.057*	
N1	0.01572 (18)	0.39651 (6)	0.53538 (7)	0.0254 (2)	
N2	0.02411 (19)	0.54214 (6)	0.73743 (7)	0.0274 (2)	
H7	0.012 (4)	0.3632 (12)	0.4892 (15)	0.056 (6)*	

### Atomic displacement parameters (Å^2^)

**Table:**

	*U*^11^	*U*^22^	*U*^33^	*U*^12^	*U*^13^	*U*^23^
O3	0.0281 (5)	0.0291 (5)	0.0406 (6)	−0.0046 (4)	0.0092 (4)	−0.0074 (4)
C1	0.0227 (5)	0.0241 (6)	0.0235 (6)	0.0010 (4)	0.0000 (4)	−0.0030 (5)
C2	0.0290 (6)	0.0267 (6)	0.0260 (6)	−0.0025 (5)	0.0063 (5)	−0.0011 (5)
C3	0.0219 (5)	0.0241 (6)	0.0159 (5)	−0.0011 (4)	0.0024 (4)	−0.0003 (4)
C4	0.0306 (6)	0.0261 (6)	0.0201 (6)	0.0041 (5)	−0.0024 (5)	−0.0032 (5)
C5	0.0229 (6)	0.0243 (6)	0.0217 (6)	−0.0005 (4)	0.0005 (4)	−0.0030 (4)
C6	0.0204 (5)	0.0221 (6)	0.0190 (5)	−0.0020 (4)	0.0017 (4)	−0.0025 (4)
C7	0.0219 (5)	0.0204 (6)	0.0183 (5)	0.0020 (4)	0.0016 (4)	−0.0030 (4)
C8	0.0268 (6)	0.0257 (6)	0.0235 (6)	0.0022 (5)	0.0003 (5)	0.0010 (5)
C9	0.0371 (7)	0.0256 (6)	0.0206 (6)	0.0020 (5)	0.0048 (5)	0.0000 (5)
C10	0.0309 (6)	0.0238 (6)	0.0260 (6)	−0.0077 (5)	0.0036 (5)	−0.0043 (5)
C11	0.0483 (8)	0.0301 (7)	0.0302 (7)	−0.0111 (6)	0.0066 (6)	0.0017 (6)
C12	0.0434 (8)	0.0365 (8)	0.0287 (7)	−0.0050 (6)	−0.0014 (6)	0.0089 (6)
O1	0.0213 (4)	0.0349 (5)	0.0343 (5)	−0.0002 (4)	0.0053 (4)	−0.0061 (4)
O2	0.0251 (5)	0.0301 (5)	0.0357 (5)	−0.0041 (4)	0.0049 (4)	−0.0140 (4)
C13	0.0235 (6)	0.0208 (6)	0.0249 (6)	−0.0005 (4)	0.0013 (4)	0.0003 (4)
C14	0.0267 (6)	0.0184 (6)	0.0226 (6)	0.0023 (4)	0.0014 (4)	0.0014 (4)
C15	0.0284 (6)	0.0225 (6)	0.0305 (6)	−0.0025 (5)	0.0000 (5)	0.0005 (5)
C16	0.0266 (6)	0.0251 (6)	0.0298 (6)	−0.0017 (5)	0.0052 (5)	0.0000 (5)
C17	0.0259 (6)	0.0241 (6)	0.0239 (6)	0.0043 (5)	0.0029 (4)	0.0015 (5)
C18	0.0406 (8)	0.0270 (7)	0.0299 (7)	−0.0053 (6)	0.0042 (5)	−0.0072 (5)
C19	0.0465 (9)	0.0346 (8)	0.0371 (8)	0.0005 (6)	0.0184 (6)	−0.0083 (6)
N1	0.0298 (5)	0.0209 (5)	0.0243 (5)	0.0017 (4)	−0.0005 (4)	−0.0022 (4)
N2	0.0318 (6)	0.0246 (6)	0.0266 (5)	0.0000 (4)	0.0061 (4)	−0.0044 (4)

### Geometric parameters (Å, °)

**Table:**

O3—H3	0.874 (15)	C11—C12	1.538 (2)
O3—H6	0.872 (15)	C11—H11A	0.9900
C1—C4	1.3937 (18)	C11—H11B	0.9900
C1—C7	1.4010 (16)	C12—H12A	0.9900
C1—H1	0.9500	C12—H12B	0.9900
C2—C9	1.3908 (19)	C13—C17	1.3649 (18)
C2—C5	1.3970 (18)	C13—C14	1.4262 (17)
C2—H2	0.9500	C13—H13	0.9500
C3—O1	1.2414 (15)	C14—N2	1.3416 (16)
C3—O2	1.2696 (15)	C14—C16	1.4189 (17)
C3—C6	1.5603 (16)	C15—N1	1.3488 (18)
C4—C9	1.3885 (19)	C15—C16	1.3625 (19)
C4—H4	0.9500	C15—H15	0.9500
C5—C7	1.3981 (17)	C16—H16	0.9500
C5—H5	0.9500	C17—N1	1.3485 (17)
C6—C7	1.5218 (16)	C17—H17	0.9500
C6—C8	1.5382 (17)	C18—N2	1.4571 (18)
C6—C10	1.5465 (16)	C18—H18A	0.9800
C8—C12	1.5345 (19)	C18—H18B	0.9800
C8—H8A	0.9900	C18—H18C	0.9800
C8—H8B	0.9900	C19—N2	1.4587 (18)
C9—H9	0.9500	C19—H19A	0.9800
C10—C11	1.5414 (19)	C19—H19B	0.9800
C10—H10A	0.9900	C19—H19C	0.9800
C10—H10B	0.9900	N1—H7	0.94 (2)
			
H3—O3—H6	105.5 (18)	C12—C11—H11B	110.5
C4—C1—C7	120.90 (12)	C10—C11—H11B	110.5
C4—C1—H1	119.5	H11A—C11—H11B	108.7
C7—C1—H1	119.5	C8—C12—C11	105.83 (11)
C9—C2—C5	120.61 (12)	C8—C12—H12A	110.6
C9—C2—H2	119.7	C11—C12—H12A	110.6
C5—C2—H2	119.7	C8—C12—H12B	110.6
O1—C3—O2	124.66 (11)	C11—C12—H12B	110.6
O1—C3—C6	119.03 (10)	H12A—C12—H12B	108.7
O2—C3—C6	116.28 (10)	C17—C13—C14	119.77 (12)
C9—C4—C1	120.23 (12)	C17—C13—H13	120.1
C9—C4—H4	119.9	C14—C13—H13	120.1
C1—C4—H4	119.9	N2—C14—C16	121.50 (12)
C2—C5—C7	120.38 (12)	N2—C14—C13	122.20 (12)
C2—C5—H5	119.8	C16—C14—C13	116.30 (11)
C7—C5—H5	119.8	N1—C15—C16	121.53 (12)
C7—C6—C8	114.25 (10)	N1—C15—H15	119.2
C7—C6—C10	113.53 (10)	C16—C15—H15	119.2
C8—C6—C10	102.06 (10)	C15—C16—C14	120.47 (12)
C7—C6—C3	105.88 (9)	C15—C16—H16	119.8
C8—C6—C3	110.33 (10)	C14—C16—H16	119.8
C10—C6—C3	110.89 (10)	N1—C17—C13	121.96 (12)
C5—C7—C1	118.48 (11)	N1—C17—H17	119.0
C5—C7—C6	121.36 (10)	C13—C17—H17	119.0
C1—C7—C6	120.11 (10)	N2—C18—H18A	109.5
C12—C8—C6	103.84 (10)	N2—C18—H18B	109.5
C12—C8—H8A	111.0	H18A—C18—H18B	109.5
C6—C8—H8A	111.0	N2—C18—H18C	109.5
C12—C8—H8B	111.0	H18A—C18—H18C	109.5
C6—C8—H8B	111.0	H18B—C18—H18C	109.5
H8A—C8—H8B	109.0	N2—C19—H19A	109.5
C4—C9—C2	119.40 (12)	N2—C19—H19B	109.5
C4—C9—H9	120.3	H19A—C19—H19B	109.5
C2—C9—H9	120.3	N2—C19—H19C	109.5
C11—C10—C6	105.25 (10)	H19A—C19—H19C	109.5
C11—C10—H10A	110.7	H19B—C19—H19C	109.5
C6—C10—H10A	110.7	C17—N1—C15	119.93 (11)
C11—C10—H10B	110.7	C17—N1—H7	120.4 (13)
C6—C10—H10B	110.7	C15—N1—H7	119.7 (13)
H10A—C10—H10B	108.8	C14—N2—C18	121.77 (11)
C12—C11—C10	106.26 (11)	C14—N2—C19	120.81 (12)
C12—C11—H11A	110.5	C18—N2—C19	117.38 (11)
C10—C11—H11A	110.5		

### Hydrogen-bond geometry (Å, °)

**Table:**

*D*—H···*A*	*D*—H	H···*A*	*D*···*A*	*D*—H···*A*
N1—H7···O2	0.94 (2)	1.72 (2)	2.6458 (15)	168 (2)
O3—H3···O2	0.87 (2)	1.93 (2)	2.7935 (14)	167 (2)
O3—H6···O1^i^	0.87 (2)	1.90 (2)	2.7634 (15)	169 (2)

Symmetry codes: (i) *x*+1, *y*, *z*.
